# Warming and Reduced Rainfall Alter Fungal Necromass Decomposition Rates and Associated Microbial Community Composition and Functioning at a Temperate–Boreal Forest Ecotone

**DOI:** 10.1111/gcb.70536

**Published:** 2025-10-09

**Authors:** Anahi Cantoran, François Maillard, Raimundo Bermudez, Artur Stefanski, Peter B. Reich, Peter G. Kennedy

**Affiliations:** ^1^ Department of Plant and Microbial Biology University of Minnesota St. Paul Minnesota USA; ^2^ Microbial Ecology Group, Department of Biology Lund University Lund Sweden; ^3^ Department of Forest Resources University of Minnesota St. Paul Minnesota USA; ^4^ College of Natural Resources University of Wisconsin Stevens Point Stevens Point Wisconsin USA; ^5^ University of Michigan School for Environment and Sustainability University of Michigan Ann Arbor Michigan USA

**Keywords:** bacteria, decomposition, fungi, necromass, reduced rainfall, warming

## Abstract

Changes in temperature and rainfall associated with altered climatic conditions are likely to significantly alter rates of soil organic matter decomposition. To determine how the combined effects of warming and drought impact the decomposition of fungal necromass, a large and fast‐cycling portion of the global soil organic carbon (C) pool, we incubated *Hyaloscypha bicolor* necromass under both ambient and altered conditions (+3.3°C air and soil warming and ~40% reduced rainfall) at the B4Warmed experiment in Minnesota, USA. We conducted two multi‐week incubations, one assessing mass loss and microbial community composition on decaying necromass after 1, 2, 7, and 14 weeks and the second characterizing the substrate utilization capacities of necromass‐associated microbial communities after Weeks 1 and 7. Warming and reduced rainfall accelerated the initial rate of necromass decay by ~20%, yet slowed overall mass loss by ~6% at the end of the 14‐week incubation. These different rates of decay over time paralleled shifting abiotic conditions, with altered plots experiencing warmer and relatively moist conditions early, but hotter and drier conditions later. The microbial community composition also varied by treatment and time, with warming and reduced rainfall stimulating fast‐growing fungi as well as fungal relative to bacterial growth overall. Additionally, the functional capacity of the microbial community also changed over time, having a higher metabolic capability to utilize C and N substrates in the altered plots early in decomposition but a lower capability later in decay. Collectively, our findings highlight a dynamic, stage‐dependent response of fungal necromass decomposition to altered climate regimes. By linking these decay dynamics to shifts in environmental conditions as well as microbial community composition and function, our study highlights the critical roles of both abiotic and biotic changes in mediating decomposition responses to climate change.

## Graphical abstract

In this study we explored how warming temperature and low water availability influenced the decomposition of dead fungal biomass. We found that warming and low water availability sped up how much the dead fungal biomass was decomposed in the first 2 weeks it was put in the field. We also found that the microbes eating it and their abilities to use different carbon and nitrogen compounds changed over time during the decomposition of the dead fungal biomass.

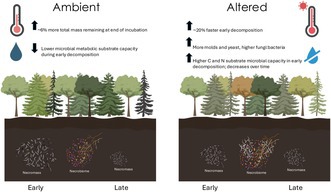

## 1 Introduction

Rising temperatures associated with elevated atmospheric greenhouse gas concentrations are altering the functioning of many ecological communities (García et al. 2018; Reich et al. 2015; Traill et al. 2010). This is particularly pertinent in ecosystems located in temperate and boreal regions, where the effects of increased temperatures are occurring most rapidly (Ito et al. 2020; Serreze et al. 2000). A critical process influenced by warming is the decomposition of organic matter, which plays a central role in carbon (C) and nutrient cycling within ecosystems (Conant et al. 2011; Davidson and Janssens 2006). Studies have shown that higher temperatures can lead to enhanced microbial activity, leading to faster breakdown of plant litter and soil organic matter (SOM) (Bond‐Lamberty et al. 2004). This, in turn, can contribute to a positive feedback loop of increased carbon dioxide emissions from soil leading to greater warming (Crowther et al. 2016). Given that soils hold twice the amount of C present in the atmosphere and vegetation combined (Lal 2004), even small changes to SOM decomposition rates may drive significant additional warming.

Along with warmer temperatures, shifting climatic conditions are also projected to make conditions drier in many temperate and boreal ecosystems (Seneviratne et al. 2006; IPCC 2023). Greater water limitation can significantly slow organic matter decomposition due to reduced microbial activity (Manzoni et al. 2012; Smith et al. 2009). Specifically, under drier conditions, microbial metabolism is constrained by lower water availability, which limits the diffusion of substrates and enzymes necessary for organic matter breakdown (Allison and Martiny 2008). Water stress thus reduces microbial growth and activity, resulting in slower decomposition rates and altered nutrient cycling (Schimel et al. 2007). As such, water availability can play an equally critical role in organic matter decomposition dynamics, potentially slowing soil carbon dioxide release and generating a negative feedback loop.

Given the contrasting trends of warming and drying trends on SOM decomposition dynamics, studying scenarios in which temperature and water availability are both manipulated in ecologically realistic ways is crucial to predicting the future functioning of temperate and boreal ecosystems. Previous research in boreal forests demonstrated that experimental warming and reduced soil moisture inhibited microbial activity and soil respiration (frequently used as proxies of organic matter decomposition) compared to ambient conditions, with a stronger effect of warming when soils were most dry (Allison and Treseder 2008). While those results suggest increased temperature and decreased moisture may additively limit microbial activity, other studies have found that simultaneous changes in both temperature and moisture may counteract one another. For example, working in a temperate forest in which temperature and rainfall were factorially altered, Schindlbacher et al. (2012) found that warming stimulated soil respiration, while reduced rainfall hindered it. However, respiration in the combined treatment was not significantly different from that in control plots. More recently, Liang et al. (2024) demonstrated that experimentally elevated temperatures stimulated soil respiration in forests at the temperate–boreal ecotone, but that this effect was dependent on soil moisture, showing less stimulation when soil moisture levels were experimentally reduced. Surprisingly, there are very few direct measures of organic matter decomposition in field experiments where temperature and moisture are both manipulated to stimulate near‐future climates, limiting our understanding of how altered abiotic conditions will influence decomposition rates and associated microbial community composition and function.

There is a growing recognition that microbial necromass contributes a significant portion to soil C pools (Fu et al. 2025), making its decomposition a critical area of study in the context of climate change (Xuan et al. 2024). Of the microbial‐derived C present in soil organic C, the necromass of fungi has been consistently observed to be 2–3 times higher than that of bacteria across ecosystems (Angst et al. 2021, 2024; Wang et al. 2021). Further, fungal necromass has also been shown to be an important source of rapidly cycling nitrogen (N) (Zhang et al. 2018), which can limit SOM decomposition rates (Averill and Waring 2018). Recent studies have indicated that, like other organic matter inputs, fungal necromass decomposition rates are sensitive to soil warming in forested ecosystems (Fernandez et al. 2019; Liu et al. 2024). Using a litter bag approach, which allows for the direct measure of necromass mass loss, Fernandez et al. (2019) found that experimentally elevated temperatures significantly increased the decomposition rates of fungal necromass in a forested peatland ecosystem, particularly in microenvironments that experienced the greatest change in water content. While those results indicate that fungal necromass decomposition is sensitive to increased temperature, possibly due to interactions with moisture availability, the unique environmental conditions of peatlands (e.g., low pH and N availability as well as low oxygen content due to a shallow water table) (Wright et al. 1992) may be unrepresentative of upland systems. Further, peatlands are known to have a strong filtering effect on microbial decomposer communities (Chen et al. 2022), so determining how more broadly distributed microorganisms are impacted by altered environmental conditions is needed to better understand how necromass decomposition and microbial community composition may be linked.

The microorganisms associated with decomposing fungal necromass, known as the fungal necrobiome, are co‐dominated by bacteria and fungi (Cantoran et al. 2023; Kennedy and Maillard 2023). The composition of the fungal necrobiome has been consistently shown to include a distinct subset of the larger soil microbial community that both changes over time (Beidler et al. 2020; Brabcová et al. 2016; Fernandez and Kennedy 2018) and is sensitive to temperature stress. For example, warming has been shown to alter the composition of the fungal necrobiome to favor fast‐growing bacteria and fungi (Maillard et al. 2022; Zhou et al. 2024). While the composition of the fungal necrobiome is increasingly well characterized, its functioning remains less frequently assessed. Fungal necromass itself is a complex mixture of proteins, polysaccharides, and phenols (See et al. 2021), requiring diverse enzymatic capabilities for breakdown. Studies examining the activity of C‐ and N‐acquiring enzymes have shown that fungal necromass can be a “hotspot” for microbial activity (Brabcová et al. 2016), although exactly which enzymes are most expressed appears to vary depending on the stage of decomposition (Maillard et al. 2021). Specifically, C acquisition may be preferentially targeted during the early stages of necromass decomposition, while N acquisition may be more common later (Maillard et al. 2021). Functional shifts also parallel changes in the composition of the fungal necrobiome community, with both ectomycorrhizal fungi and oligotrophic bacteria being increasingly abundant at the later stages of decay (Maillard et al. 2021, 2022). The extent to which the above enzyme activity patterns are impacted by rising temperatures and declining soil moisture is unknown, representing a critical knowledge gap in efforts to predict microbial processes in a changing climate.

In this study, we assessed the dynamics of fungal necromass decomposition at a boreal–temperate forest ecotone in North America, which is currently experiencing significant climate‐related changes in forest functioning and composition (Fisichelli et al. 2014; Reich et al. 2022). We measured fungal necromass mass loss over a 14‐week period in open‐air experimental plots with increased temperature and decreased rainfall relative to plots with ambient temperature and precipitation. We also characterized the structure of microbial communities associated with decomposing fungal necromass in both plot types at 4 time points during the 14‐week incubation, as well as the soil microbial communities at the beginning of the incubation. Further, we deployed a second set of fungal necromass samples in the same plots to assess the functional capacity of the fungal necrobiome community using Biolog “EcoPlate” plates at early (1 week) and later (7 weeks) stages of decomposition. We tested the following three hypotheses: (1) increased temperature and reduced rainfall will stimulate the decomposition of fungal necromass, similar to the enhanced soil respiration rates observed in this treatment previously (Liang et al. 2024); (2) increased temperature and reduced rainfall will significantly alter the composition of the fungal necrobiome, particularly favoring fast‐growing bacteria and fungi, and (3) increased temperature and reduced rainfall will favor microbial communities with enhanced capacity to utilize simple forms of C early in decomposition due to the relatively low C:N of necromass, but then transition to a higher capacity to use N‐containing substrates and more complex forms of C later in decomposition as labile C is depleted.

## 2 Methods

### 2.1 Field Site and Experimental Design

This study was conducted at the University of Minnesota Cloquet Forestry Center in Cloquet, Minnesota, USA, in one of two sites for the B4Warmed (Boreal Forest Warming at an Ecotone in Danger) experiment (46°40′46″N, 92°31′12 W). This site has a mean annual air temperature of 4.5°C and mean annual precipitation of 807 mm (Rich et al. 2015). Climate projections for this region over the 21st century predict elevated air temperatures as well as increased annual precipitation, although the summer months will become drier and feature longer periods of extended soil water deficit (Liess et al. 2022). The experiment is composed of 3‐m diameter circular plots, with factorial experimental warming (ambient and +3.3°C) heated using infrared lamps and soil heating cables and reduced mid‐summer rainfall (ambient and 40% reduction) (see Rich et al. 2015; Stefanski et al. 2020, for further details). For our study, we only utilized two of the four treatments: the ambient temperature and ambient rainfall treatment (hereafter referred to as ambient) and the +3.3°C and reduced rainfall treatment (hereafter referred to as altered). The experimental layout consists of three blocks located ~20 m apart, each of which contains a single plot for each of the aforementioned treatments. All the plots had an open overstory and contained a mixture of randomly assigned 19 native Minnesota tree species ranging in age from 2 to 4 years old.

### 2.2 Site Moisture and Temperature Measurements

Soil volumetric water content (cm^3^ H_2_O/cm^3^ soil) from 0 to 20 cm of each plot was measured hourly using a Campbell Scientific CS‐616 probe inserted into the soil at a 45° angle (Rich et al. 2015). Aboveground temperature was measured at mid‐canopy height (i.e., roughly the average for all planted tree seedlings in each plot) with thermocouples suspended in acrylic blocks imitating a leaf surface. Belowground temperature was measured using sealed thermocouples at a 10 cm soil depth, each monitored continuously (logged every 1 min and averaged to 60 min intervals) (Rich et al. 2015).

### 2.3 Necromass Production and Field Incubation

Fungal necromass from *Hyaloscypha bicolor* (formerly *Meliniomyces bicolor*) was generated by growing mycelium on potato dextrose agarose (PDA) for 3 weeks, after which plugs were taken and transferred to PD broth (pH 5) and incubated on orbital shakers at 150 rpm for 30 days at 25°C. Mycelium was collected, washed, homogenized, and freeze‐dried for 3 days at −50°C to create the fungal necromass (see Pérez‐Pazos et al. 2024 for details). *H. bicolor* was used because it is common mycorrhizal fungus found in high latitude forests (Grelet et al. 2009; Kohout et al. 2018) and the composition of its necrobiome has been well characterized in previous studies (Fernandez et al. 2019; Fernandez and Kennedy 2018; Pérez‐Pazos et al. 2024). The *H. bicolor* necromass used in this experiment was identical to that used by Pérez‐Pazos et al. (2024), having a C% of 46.64, N% of 4.51, and a C:N of 10.3. Necromass was added to 53‐μm nylon mesh bags (R510, ANKOM Technology, NY, USA) and heat sealed, with a total of 60 mg (±1 mg) of necromass added for 24 bags for the first time point (after 1 week, *n* = 24) and 100 mg (±1 mg) of necromass was added for the subsequent time points (*n* = 72) resulting in a total of 96 necromass bags.

Necromass bags were incubated ~5 cm deep in soil in clusters of the four quadrants of each plot (Figure S1). Four separate bags were adjacently placed in each cluster (within 20 cm), for a total of 16 necromass bags' incubation in each plot (Figure S1). Necromass bags were deployed on June 28, 2023, and incubated for a total of 1, 2, 7, and 14 weeks. Harvests entailed the removal of one bag from each quadrant at each time point, transporting the bags back to the University of Minnesota and storing them at −20°C. The following day, the necromass was scraped with a sterile spatula and moved to a sterile 1.5 mL microcentrifuge tube and stored at −20°C. To capture the initial microbial community composition at the different sites, small “Whirl‐Pak” sterilized bags (whirl‐pak.com) were filled with soils the day of deployment from an adjacent spot where bags were deployed for each quadrant in each plot (*n* = 24), transported back to the University of Minnesota and stored at −80°C until further analyses.

### 2.4 Necrobiome Substrate Utilization

A separate necromass incubation was deployed on August 16, 2023, using the same *H. bicolor* necromass. Bags were incubated for 1 and 7 weeks to represent early and late decay phases. Bags were deployed at two blocks in for each of the ambient and altered conditions. Three bags were incubated in clusters in three out of four quadrants of each plot in two out of the three blocks (blocks D and E, *n* = 12 per plot). The necromass bags were pooled for each plot after 1 and 7 weeks of incubation to obtain a homogenous representation of the necrobiome communities. A total of 200 mg (±20 mg) of necromass from the pooled bags were aliquoted and then suspended in sterile distilled water (1/20 mv) in a 15 mL conical tube and vortexed for 2 min. In addition, a set of bags was also deployed in the remaining quadrant for plots in blocks D and E, and in a single quadrant of plots located in block F to microbial community composition and mass loss after Week 1 and 7.

To quantify microbial community substrate utilization, EcoPlates (Biolog, Hayward, CA, USA) were used for each block and treatment (*n* = 4) from the pooled necromass slurries. The supernatant was diluted to 10^−1^ and 100 μL were suspended into the microplate wells and then incubated at 20°C in the dark. The absorbance was measured with a SpectraMax i3x Multi‐Mode Microplate Reader after 120 h at a wavelength of 590 nm. The measurements were done in triplicate with three blanks (water) per plate. The OD values were corrected by subtracting the OD of the blanks from the OD of each substrate well, with negative values converted to zero.

Each substrate was grouped into a substrate type category: Amines, Amino Acids, Carbohydrates, Carboxylic Acids, Phenols, and Polymers, respectively (Table S1). The substrate average well color development (AWCD) was calculated from the final time point collected (120 h) using the following equation: AWCD = ∑OD_i_/*N*, where OD_i_ is the corrected OD value of the substrates within a substrate category and *N* is the number of substrates in that respective category (Feigl et al. 2017).

### 2.5 Molecular Analyses

Bacterial and fungal communities on necromass as well as in soils collected at the beginning of the incubation were identified using high throughput sequencing (HTS). Total gDNA was isolated from necromass (10 ± 1.5 mg) and soil (25 ± 1.5 mg) using the DNeasy PowerSoil Extraction Kit (QIAGEN). Extractions followed manufacturer's instructions, with the addition of three glass 2 mm beads and a bead beating step for 30 s. 16S (bacterial) and ITS2 (fungal) DNA amplicons were generated from extracted DNA at the University of Minnesota Genomics Center (UMGC, Minneapolis, MN). 16S amplicons were generated by a previously published method optimized by the UMGC (Gohl et al. 2016) using a dual‐indexed PCR to amplify the V4 region of the bacterial rDNA locus using KAPA HiFi polymerase. ITS2 amplicons were generated using the same dual‐index technique with a fungal primer set targeting the ITS2 region of the fungal rDNA locus (forward primer, FSeq2, sequence: TCGATGAAGAACGCAGCG; reverse primer, RSeq) (Heisel et al. 2015) and KAPA HiFi Hotstart plus dNTPs (Roche) for amplification. Positive controls were also amplified; the Microbial Community Standard (ZymoBIOMICS) for bacteria and synthetic ITS mock community for fungi (Palmer et al. 2018). Additionally, negative controls were included with no DNA template added. For PCR, 25 cycles were used for 16S and ITS2 amplicon generation. Samples were sequenced on a full NextSeq run (2 × 300 bp Illumina chemistry), with 16S and ITS2 amplicons include in the same run.

Raw sequence files were initially processed with cutadapt (Martin 2011) to identify all sequence reads with primers and sequencing adapters and remove both. The remaining sequences were filtered, denoised, and merged using the DADA2 algorithm (Callahan et al. 2016) using recommended parameters for 16S (maxN = 0, maxEE = c(2, 4), truncQ = 2) and ITS (maxN = 0, maxEE = c(2, 2), truncQ = 2, minLen = 50). Amplicon sequence variants were assigned taxonomy using the SILVA (v138.1, Quast et al. 2012) and UNITE (v9, Abarenkov et al. 2024) databases for 16S and ITS ASVs, respectively. All forward and reverse .fastq files were deposited in the NCBI Short Read Archive under BioProject numbers PRJNA1291239 (Bacteria) and PRJNA1291730 (Fungi).

For each ASV table, sequence reads present in the negative controls were subtracted from the read abundances present in the necromass samples. The mock communities were used to determine a low sequence read cut‐off to account for tag switching (sensu Carlsen et al. 2012) across samples (i.e., all values below 15 and 10 reads in the bacterial and fungal ASV tables were zeroed, respectively). Sequence read–ASV curves were plotted for bacteria and fungi separately, with both showing that nearly all samples, particularly on necromass, reach saturation by 1000 reads (Figure S7). To assign bacterial trophic mode lifestyles, we classified ASVs belonging to Betaprotebacteria, Gammaproteobacteria, Alphaproteobacteria as copiotrophic, and Acidobacteria, Actinobacteria, and Deltaprotebacteria as oligotrophic based on Trivedi et al. (2018). For fungi, we classified ASVs belonging to the Eurotiales, Hypocreales, Morteriellales, Mucorales, Saccharomycetales, Tremellales, and Sporidiales as molds and yeasts (reflecting a r‐strategist lifestyle) based on Sterkenburg et al. (2015).

Total genomic DNA from the samples used for microbial substrate utilization was also assessed with quantitative real‐time PCR (qPCR). The abundance of total bacteria and fungi was assessed using 16S rRNA primers 1401F/968R (Cébron et al. 2008) and 18S rRNA primers FR1/FF390 (Prévost‐Bouré et al. 2011), respectively. The qPCR reactions were performed in a 20 μL volume containing 2 μL of DNA, 10 μL of SYBR Green dye, 6.4 μL molecular grade water, and 0.8 μL of each primer at 10 μM. Amplification conditions consisted of 5 min at 95°C, followed by 40 cycles of 20 s at 95°C, 30 s at the primer‐specific annealing temperatures (56°C and 50°C for 16S and 18S rRNA, respectively), and 60 s at 72°C on a StepOne Realtime PCR machine (ThermoFisher Scientific, Waltham, MA, USA). For each sample, two independent qPCR reactions were performed for bacteria and fungi. The qPCR values are reported as gene log copies per gram of necromass.

### 2.6 Statistical Analyses

Given the well‐established nonlinear nature of fungal necromass decomposition (See et al. 2021), we fitted the proportion of remaining necromass against incubation time (days) in the ambient versus experimental plots (i.e., those with increased temperature and decreased precipitation) using asymptotic and double‐exponential decay models. We found that the best fitting model, selected using Akaike's information criteria (AIC), was asymptotic. The mean AIC value for the asymptotic models of mass remaining was 0.14 (±0.13 SE), indicating strong parameter estimates were generated despite a limited number of sampling times (*N* = 4). To obtain values for *k* (the initial rate of decay) and *A* (the stable fraction remaining), asymptotic models were run on every set of samples, with each set representing a group of necromass bags in each quadrat. Generalized linear models were then performed (using the “nlme” package) on model‐derived *k* and *A* values, with treatment as the explanatory variable and cluster nested within block as random effects. (Cluster was included in the models as a nested random effect because each cluster's surrounding tree community was distinct across all plots—see Figure S1 for details). No decay models or statistical tests were applied to the necromass bags deployed in the 7‐week incubation because the replication to assess necromass mass loss was very low (*n* = 3).

To assess changes in microbial community composition on fungal necromass, we first center log transformed bacterial and fungal sequence reads using the “CoDaSeq” and “zcompositions” packages, following the recommendation of Gloor et al. (2017). We then calculated Aitchison's distances between all sample pairs and used PCA to visualize differences by treatment and time. Significant differences by treatment, time, and their interaction were determined using PERMANOVAs (using the adonis2 function), with the “vegan” package. The PERMANOVAs included block as a random factor. We also performed differential abundance analyses on the same microbial communities using ALDEx2. For the 15 most abundant bacterial and fungal genera, Cohen's *d* effect size was calculated as the mean center log transformed read abundance on necromass when incubated in altered plots minus the mean center log transformed read abundance when incubated in ambient plots divided by the pooled standard deviation of center log transformed read abundance on necromass incubated in either plot type. Samples were binned into early (Days 7 and 14) and late (Days 49 and 98) stages of decomposition for the Cohen's *d* calculations.

To test for differences in microbial community substrate utilization in the 7‐week incubation, three‐way ANOVAs were conducted to test for differences by compound category, treatment, incubation time, and their interactions. Finally, to determine whether bacterial and fungal log copies per gram of necromass differed across treatments and incubation time in the substrate utilization assay, two‐way ANOVAs were conducted. Post hoc analyses for ANOVAs were run using Tukey's HSD test using the “stats” package, and compact letter displays were obtained using the “agricolae” package. All statistical analyses and data visualization were conducted in R version 4.2.3 (R Core Team 2025).

## 3 Results

### 3.1 Treatment Effects on Climatic Conditions and Soil Microbial Communities

Average temperatures in the altered and ambient plots during the duration of the 14‐week incubation were 20.0 ± 1.6 (mean ± 1 SD) and 17.6 ± 1.8 (°C), respectively. The average volumetric water content (VWC) in the altered and ambient plots was 0.12 ± 0.04 and 0.17 ± 0.04 cm^3^ H_2_O/cm^3^ soil, respectively. Weekly averages for both variables varied over the length of the experiment due to seasonal fluctuations in air temperature and precipitation, although the differences between treatments stayed generally consistent (Figure S2). The soil microbial communities were also significantly different between altered and ambient plots for both bacteria and fungi (Bacteria: PERMANOVA *p* = 0.005; Fungi: PERMANOVA *p* = 0.001, Figure S3). The altered plots were dominated by the bacterial genera *Bradyrhizobium*, *Candidatus Udaeobacter*, and *Acidothermus* and the fungal genera *Penicillium*, *Podila*, and *Trichoderma*. Ambient plots were dominated by the bacterial genera *Bradyrhizobium*, *Candidatus Udaeobacter*, and *Gaiella*, and the fungal genera *Podila*, *Solicoccozyma*, and *Trichoderma* (Table S7). Despite these taxonomic shifts, the proportion of copiotrophic bacteria or fast‐growing molds and yeasts did not significantly differ across treatments (Bacteria: *χ* ^2^ = 0.140, df = 2, *p* = 0.932; Fungi: *χ* ^2^ = 0.796, df = 1, *p* = 0.372) (Figure S3).

### 3.2 Necromass Mass Loss

**FIGURE 1:**
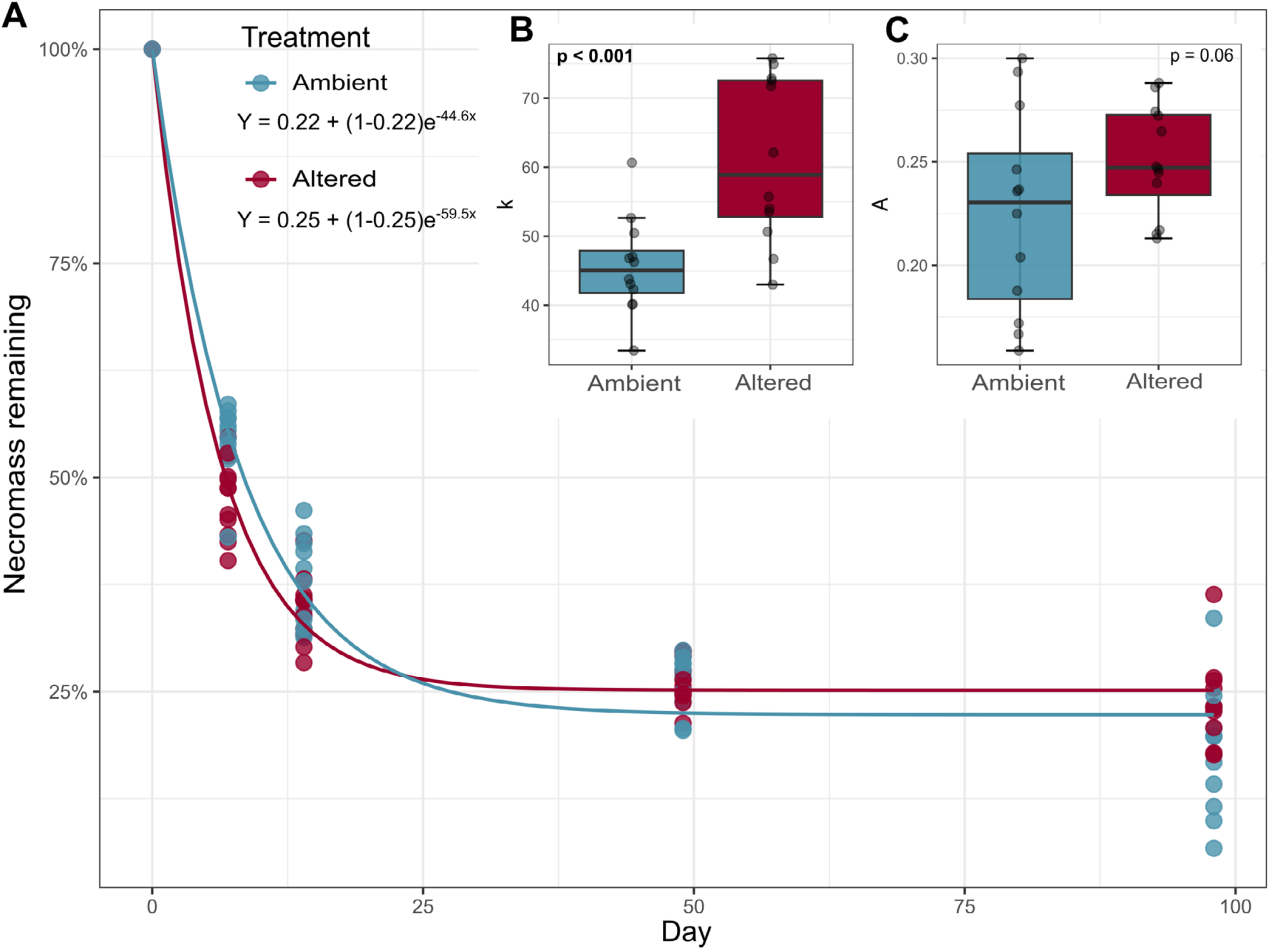
Fungal necromass mass loss during the 14‐week field incubation. (A) Asymptotic decay model of the percent necromass remaining over time for ambient (blue) and altered (red) conditions. (B) Decay rate, *k*, and (C) Asymptotic fraction, *A*, sizes for the ambient and altered conditions. Values were obtained independently for each set of replicates (*N* = 12). ANOVA *p*‐values are presented for *k* and *A* values, with treatment as the explanatory variable.

The decomposition of fungal necromass occurred rapidly, with nearly 70% of the initial mass being lost in the first 2 weeks regardless of plot treatment (Figure 1). During this initial decay stage, however, there was a significant difference in rate (*p* < 0.001), with *k* values being approximately 20% higher on average in the altered plots compared to the ambient plots (Figure 1B). Mass loss in both treatments declined after 4 weeks, with the A fraction being approximately 6% higher on average in the altered plots compared to the ambient plots. This latter difference was marginally significant (*p* = 0.061) (Figure 1C). The mass loss patterns for the additional bags that were incubated during the substrate utilization assay showed limited differences at Day 7, but consistently higher necromass mass remaining in the altered plots at Day 49 (Figure S4).

### 3.3 Necrobiome Community Composition

**FIGURE 2:**
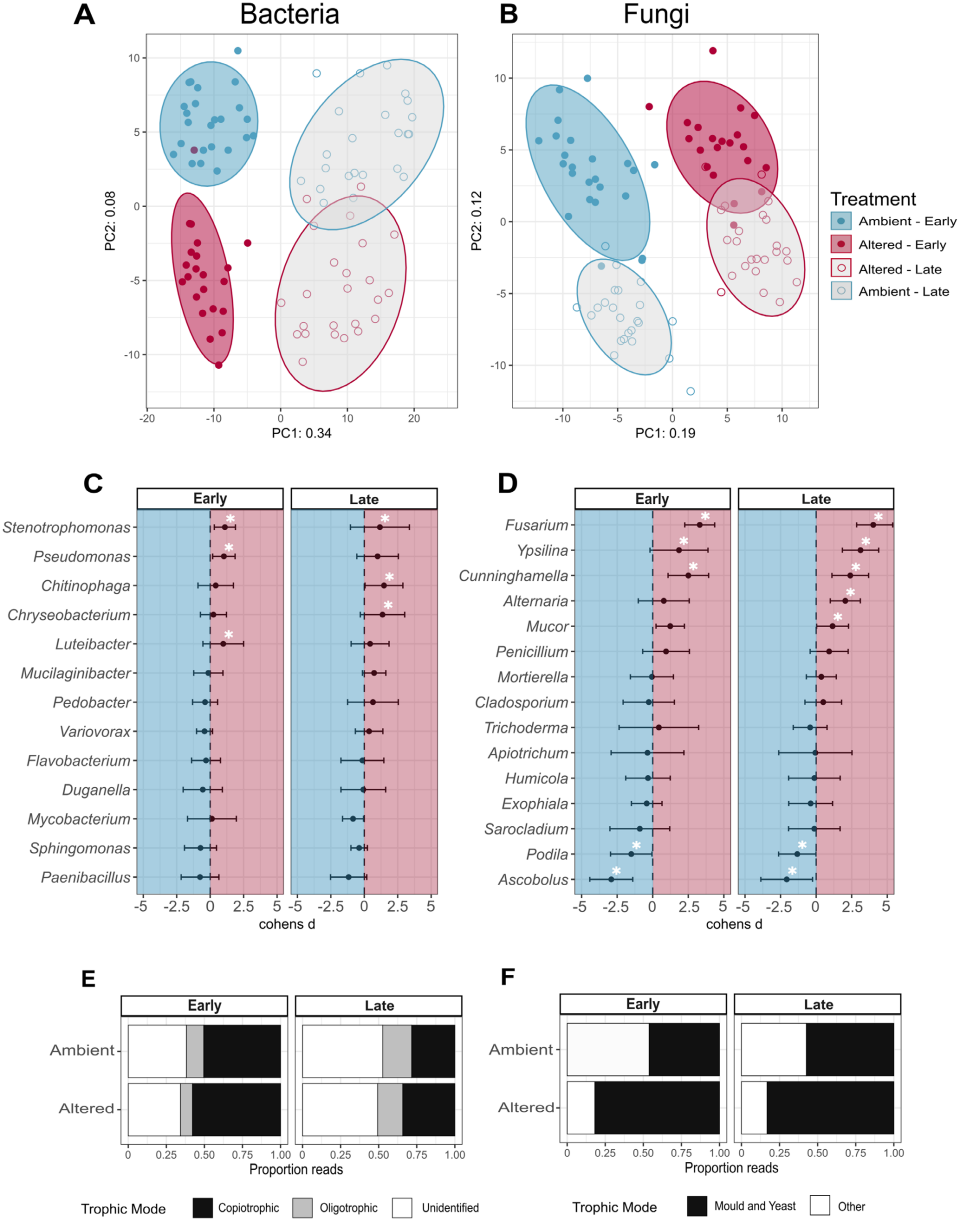
Microbial communities on decaying fungal necromass during the 14‐week field incubation. Principal Components Analysis (PCA) for bacterial (A) and fungal (B) communities on necromass. Microbial communities for the ambient conditions are represented in blue and altered conditions are in red. Different time frames are represented as “Early” for necromass incubation at Days 7 and 14, and “Late” for necromass incubation at Days 49 and 98. Lighter shades of color and open circles represent the late period. Cohen's *d* values for the 15 most abundant bacterial (C) and fungal (D) genera. White asterisks represent whether that genus was significantly differentially abundant based on ALDEx2 for each time frame. Error bars represent ±1 standard deviation. Proportion of sequence reads of bacteria (E) and fungi (F) found on necromass classified by their trophic modes across time periods and treatments. Trophic modes for bacteria include copiotrophic (black), oligotrophic (gray), and unidentified (white), while trophic modes for fungi include mold and yeast (black) and other (white).

The composition of the bacterial and fungal communities on decaying fungal necromass differed significantly by treatment and over time (Bacteria: PERMANOVA *p* = 0.005, Fungi: PERMANOVA *p* = 0.005) (Figure 2A,B). In the altered plots, fungal necromass was most highly colonized by bacteria in the genera *Luteibacter*, *Pseudomonas*, and *Stenotrophomonas*, and fungi in the genera *Fusarium*, *Mucor*, and *Trichoderma*, while in the ambient plots necromass was most highly colonized by bacteria in the genera *Mucilaginibacter*, *Paenibacillus*, and *Pseudomonas* and fungi in the genera *Mucor*, *Podila*, and *Humicola*. Many bacterial and fungal genera exhibited significant differential abundances between the ambient and altered plots, although those differences also depended on the stage of decomposition (Figure 2C,D; Table S7). Warming and reduced rainfall increased the proportion of copiotrophic bacteria and fungal molds and yeasts in the necromass communities compared to ambient plots. However, this increase was only significant in fungal communities early in the decomposition process (Bacteria Early: *χ* ^2^ = 0.282, df = 2, *p* = 0.869; Bacteria Late: *χ* ^2^ = 0.191, df = 2, *p* = 0.909; Fungi Early: *χ* ^2^ = 4.245, df = 1, *p* = 0.020; Fungi Late: *χ* ^2^ = 2.462, df = 1, *p* = 0.117) (Figure 2E,F).

### 3.4 Necrobiome Substrate Utilization

**FIGURE 3:**
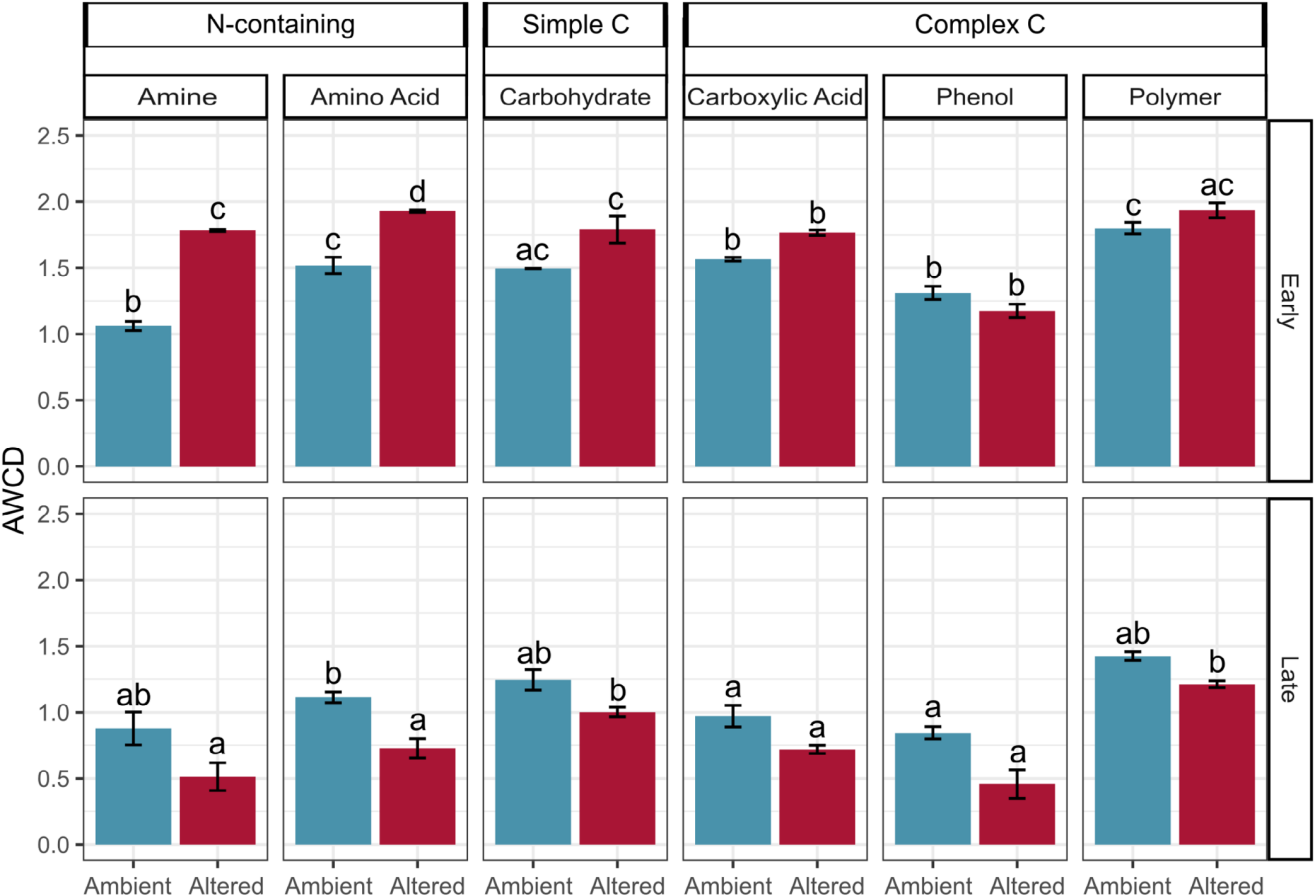
Average well color development (AWCD) for each substrate type from necromass‐associated microbial communities assayed in the 7‐week incubation. Top row represents the early (Day 7) incubation and bottom row is the later (Day 49) incubation. Columns from left to right represent different classes of substrates; Amine (*n* = 2), Amino Acid (*n* = 6), Carbohydrate (*n* = 10), Carboxylic Acid (*n* = 7), Phenol (*n* = 2), and Polymer (*n* = 4). See Table S1 for detailed substrate grouping. Each bar represents the mean of the total number of substrates that fall under each class, with error bars representing ±1 standard deviation. Significant differences between treatments across the two incubation time periods from Tukey's HSD test are indicated with different letters.

The AWCD values obtained from the substrate utilization incubations were significantly different across substrate (*p* < 0.001), time (*p* < 0.001), and all interactions (*p* < 0.010) (Figure 3). AWCD values were higher for microbial communities from the altered plots early in decomposition for 6 of the 7 categories, although only significant for amines and amino acids. Later in decomposition, AWCD values were generally reversed, with all seven categories being higher in the control plots. Amines, amino acids, and phenols all had significantly higher AWCD values for microbial communities from the control plots at this later time point, while no other categories were significantly different. In addition to the general reversal in AWCD values across treatments over time, the values later in decomposition were also consistently lower than early in decomposition, particularly in the altered plots.

During the full 14‐week incubation, the microbial communities were significantly influenced by both treatment and time. This was also true for the bacterial and fungal community composition in the 7‐week incubation (Figure S5). Though microbial community composition significantly differed on necromass between the 14‐week incubation and the 7‐week incubation (Table S3), the most abundant bacteria and fungi were shared across incubations. For example, bacteria such as *Flavobacterium*, *Pseudomonas*, and *Stenotrophomonas*, and fungi such as *Fusarium*, *Mucor*, and *Trichoderma*, remained among the top 15 most abundant taxa in both incubations across plot types (Table S7). In the 7‐week incubation, the bacterial communities present on necromass had similar abundances across treatments and time periods (based on 16S copy number counts) (*F*_1,16_ = 0.056, *p* = 0.815, Figure S6A), while the fungal communities had significantly higher abundances on necromass in the altered plots compared to ambient plots at both time periods (*F*_1,16_ = 6.972, *p* = 0.0178, Figure S6B). Additionally, the fungal to bacterial (F:B) ratios differed significantly by treatment (*F*_1,16_ = 20.144, *p* = 0.0004) and its interaction with time (*F*_1,16_ = 12.284, *p* = 0.003, Figure S6C), indicating a shift toward greater fungal‐dominated necromass decomposition under warming and reduced rainfall.

## 4 Discussion

Our study revealed a temporally dynamic response of fungal necromass decomposition to warming and reduced rainfall at the temperate–boreal forest ecotone. Partially consistent with our first hypothesis, we saw that warming and reduced rainfall did significantly accelerate the early rates of necromass decomposition, which parallels the increased soil respiration rates previously observed in the same treatment in this study system (Liang et al. 2024). At the same time, our result of ~6% greater necromass mass remaining under warming and reduced rainfall contrasts with our hypothesis of consistent increased decomposition in the altered plots. This latter finding is also different from the results of a previous field study in Minnesota, where warming alone enhanced fungal necromass loss in later stages (12–104 weeks; Fernandez et al. 2019). However, our overall findings align with a recent global meta‐analysis indicating no significant impact of either increased temperature or decreased moisture on fungal necromass stocks (Hu et al. 2023), as the early stimulation but later inhibition of decomposition observed would result in limited overall change in total necromass stocks for altered compared to ambient plots. Since we only measured mass loss (a stock output) though, future research that integrates fungal necromass inputs in this system will be critical for fully understanding the complex dynamics of fungal necromass stocks under climate change.

We suspect that the stage‐dependent response in decomposition likely arose from three factors. First, because our experiment occurred under field conditions, abiotic factors shifted throughout the study period. As early‐stage decomposition coincided with wetter soils (Figure S2), warming likely favored fast‐growing microbial taxa that could capitalize on more labile forms of necromass C, and climate manipulations did not drive soil moisture to levels where they would overwhelm beneficial thermal effects. Conversely, the deaccelerated mass loss in the altered plots compared to the ambient plots occurred when overall soil moisture levels were generally lower, such that the effects of increased temperature and reduced rainfall likely made growing conditions for microbes less favorable in that treatment (Schimel 2018). A second factor contributing to our stage‐dependent results was likely the changing chemical composition of the necromass itself. As necromass decomposes, it becomes enriched in recalcitrant compounds (Ryan et al. 2020; See et al. 2021), which are more resistant to microbial breakdown. This increasing recalcitrance, along with greater soil water limitation in the later stage of our experiment, may have further slowed decomposition rates, offsetting the initial gains observed under warmer and wetter conditions. The third factor that likely differentially influenced the two stages of decomposition was the functional capacity of the microbial community. As demonstrated in our 7‐week incubation, the necromass‐associated microbial communities in the altered plots had consistently higher abilities to utilize a range of C and N sources, including both simple and complex C forms, during the early stages of decomposition compared to those from the ambient plots. This utilization capacity, however, reversed in the later stages of decomposition, with the communities from the ambient plots having greater C and N degradation abilities than those from the altered plots. Notably, we suggest that the three aforementioned factors—shifting abiotic conditions, altered necromass substrate availability, and changed microbial community functional capacity—likely acted synergistically to drive the stage‐dependent response observed. Taken together, our findings highlight the importance of considering stage‐dependent decomposition dynamics and microbial community responses when assessing the impacts of climate change on forest ecosystems.

The shifts that were observed in soil bacterial and fungal community composition between the experimentally altered and ambient plots align with previous research in this study system (Fernandez et al. 2023; Van Nuland et al. 2020). While the dominant bacterial and fungal genera differed between treatments, overall relative abundances of copiotrophic bacteria, as well as fungal molds and yeasts, remained relatively constant in the soil. However, on necromass, we observed sizable increases in fungal molds and yeasts in the warmed plots, particularly during the early stages of decomposition. Overall, 8 of the 15 most abundant fungal genera were classified as molds or yeasts (Figure 2), although some, such as *Ascobolus* and *Podila*, showed significantly greater abundances in the ambient plots. Similarly, 10 of the 15 most abundant bacterial genera were classified as copiotrophic (Figure 2). Given the low C:N ratio of fungal necromass relative to other organic matter inputs (Beidler et al. 2020; DeLancey et al. 2024), it is perhaps unsurprising that fast‐growing bacteria and fungi are a dominant part of the necrobiome under conditions highly favorable to growth, which supported our second hypothesis.

Consistent with other necromass decomposition studies (Cantoran et al. 2023), we observed significant shifts in the composition of the necrobiome community over time regardless of plot treatment. Interestingly, the extent of temporal change in the community was greater in the ambient plots for both bacteria and fungi. This could be due to the more rapid initial decomposition of necromass under the combined stress treatment, potentially hastening microbial succession (Tang et al. 2023). Alternatively, the altered plots may have filtered the necrobiome community, limiting compositional changes over time, possibly due to increased network complexity (Yuan et al. 2021). Regardless of the mechanism, these results also support our second hypothesis and suggest that warmer and drier climates may favor a necrobiome increasingly dominated by rapidly growing, opportunistic taxa.

Our 7‐week incubation provided further insights into the potential functional capacity of the necrobiome community across early and later decomposition stages. As noted above, consistent with the rapid initial mass loss observed in the 14‐week incubation, the Week 1 necrobiome community showed significantly higher overall activity than the Week 7 community, mirroring patterns observed for C‐ and N‐acquiring enzymes in other studies (Brabcová et al. 2016). Contrary to our third hypothesis, however, this difference was most pronounced for N‐containing substrates (amino acids and amines), suggesting that fungal necromass may be a particularly important source of labile N. This aligns with previous findings demonstrating rapid N incorporation by bacteria and fungi during fungal necromass decomposition (Maillard et al. 2023; Narayanan et al. 2025) and with observations that most of the N release from fungal necromass occurs within the first 9 days, especially at higher temperatures (Wang et al. 2020).

The reversal in the functional potential of the necromass‐associated microbial communities at Week 7 coincided with a significant shift in community composition, suggesting that later colonizing microbes had a reduced capacity to utilize necromass resources. This could reflect a greater allocation of resources to cellular maintenance under the more stressful conditions of the altered plots (Romero‐Olivares et al. 2019). However, because the assay represents an aggregate measure, our findings do not clearly represent how specific microbial taxa actively metabolize necromass over time and in different environmental conditions. Further research, such as culture‐based tests and transcriptomic analyses on early and later colonizing taxa, is needed to disentangle the relative contributions of intrinsic microbial capacities versus environmental effects. Contrary to expectations based on earlier findings (Brabcová et al. 2016), bacterial abundance remained consistent between the two time points, indicating that necromass remains a hotspot of bacterial activity even in later decay stages. Furthermore, the higher F:B ratios across treatments and time points suggest that fungi may be more responsive to warming and reduced rainfall than bacteria (Zhou et al. 2024).

Our findings on fungal necromass decomposition have implications for understanding how climate change may alter the dynamics of soil C and N pools. The recalcitrant fraction of necromass, A, which is enriched in high molecular weight compounds (Lavallee et al. 2020; Ryan et al. 2020), likely represents a source of organic matter that could contribute to the particulate organic matter (POM) pool. Seeing a larger A fraction in the altered plots suggests that later‐stage necromass decomposition under warming and reduced rainfall may increase soil POM pools. However, this difference by plot type was only marginally significant (*p* = 0.061), indicating that methods such as size fractionation and chemical analyses are needed to better clarify the magnitude at which fungal necromass contributes to the POM pool under climate change. Additionally, our result is based on the necromass of a single species, and A fraction size is strongly influenced by cell wall melanin content (Fernandez et al. 2019). Specifically, melanin content increases the A fraction size (See et al. 2021), and since melanin is often produced by fungi in greater amounts under stressful conditions (Koide et al. 2014), warming and reduced rainfall may impact the initial chemistry of the whole necromass pool. As such, future research should investigate necromass from species with diverse chemistries to assess the generality of A fraction findings.

In contrast to the A fraction, the ~20% increase in the initial rate of necromass decay under warmer and drier conditions could potentially stimulate mineral‐associated organic matter (MAOM) C formation. This is because the rapid breakdown of necromass releases low molecular weight compounds (See et al. 2021; Maillard et al. 2023), which could readily bind to mineral surfaces (Lavallee et al. 2020). However, this potential increase in the MAOM pool may be offset by two counteracting factors. First, fast‐growing microorganisms, which are favored under warmer conditions, generally have lower carbon use efficiency (CUE) (Domeignoz‐Horta et al. 2020). This means they respire a greater proportion of the necromass C, potentially reducing the amount incorporated into the soil. Second, warming can slow MAOM formation (Zhao et al. 2024). Therefore, the net effect of increased necromass input on MAOM stocks remains unclear, and directly quantifying the POM and MAOM C and N pools in combination with measures of fungal hyphal production is needed to assess the cumulative effects of altered necromass decomposition on soil C and N stocks. Finally, the observed shift toward a more fungal‐dominated necrobiome under altered conditions could also potentially influence soil C persistence. Fungi tend to have a higher CUE than bacteria (Soares and Rousk 2019), and if fungi remain more active under experimental warmed and reduced rainfall conditions (Zhou et al. 2024), more C may be retained in soil (Canarini et al. 2024; Keiblinger et al. 2010). This possibility is supported by other studies demonstrating that soils with high F:B ratios often have higher C stocks (Bailey et al. 2002; Malik et al. 2016).

## 5 Conclusion

Our field‐based study demonstrates that fungal necromass decomposition in the boreal–temperate ecotone responds to altered climatic conditions in a rapid yet stage‐dependent manner. This ecotone is particularly vulnerable to climate change due to the rapid shifts in temperature and precipitation patterns occurring in these regions (Ito et al. 2020). Our findings show that while warming combined with reduced rainfall initially accelerated decomposition, it also resulted in less total mass loss due to decelerated decomposition over time. By linking these decay dynamics to shifts in microbial community composition and function, our study highlights the critical role of microbial communities in mediating decomposition responses to climate change. Further, by elucidating the links among altered climate regimes, microbial communities, and necromass decomposition dynamics, we emphasize the need for further research to fully assess the consequences of these changes at the boreal–temperate ecotone and similar transitional zones.

## Author Contributions

**Anahi Cantoran:** data curation, formal analysis, investigation, methodology, visualization, writing – original draft. **François Maillard:** methodology, visualization, writing – review and editing. **Raimundo Bermudez:** data curation, resources, writing – review and editing. **Artur Stefanski:** resources. **Peter B. Reich:** resources, writing – review and editing. **Peter G. Kennedy:** conceptualization, funding acquisition, methodology, supervision, validation, visualization, writing – review and editing.

## Conflicts of Interest

The authors declare no conflicts of interest.

## Supporting information


**Tables S1–S7:** gcb70536‐sup‐0001‐Tables.xlsx.

**Figures S1–S7:** gcb70536‐sup‐0002‐FigureS7.pdf.

## Data Availability

The data that support the findings of this study are openly available in Dryad at https://doi.org/10.5061/dryad.rn8pk0pp6. Code for this study is publicly available at https://zenodo.org/records/17167747.
