# Supplementary material for: Warming and Reduced Rainfall Alter Fungal Necromass Decomposition Rates and Associated Microbial Community Composition and Functioning at a Temperate–Boreal Forest Ecotone
Source: Glob Chang Biol. 2025 Oct 9;31(10):e70536. doi: 10.1111/gcb.70536 (PMC12509255; doi:10.1111/gcb.70536)
Supplement: Supplementary file 2 — Figures S1–S7: gcb70536‐sup‐0002‐FigureS7.pdf. [file GCB-31-e70536-s001.pdf]

SUPPLEMENTAL FIGURES

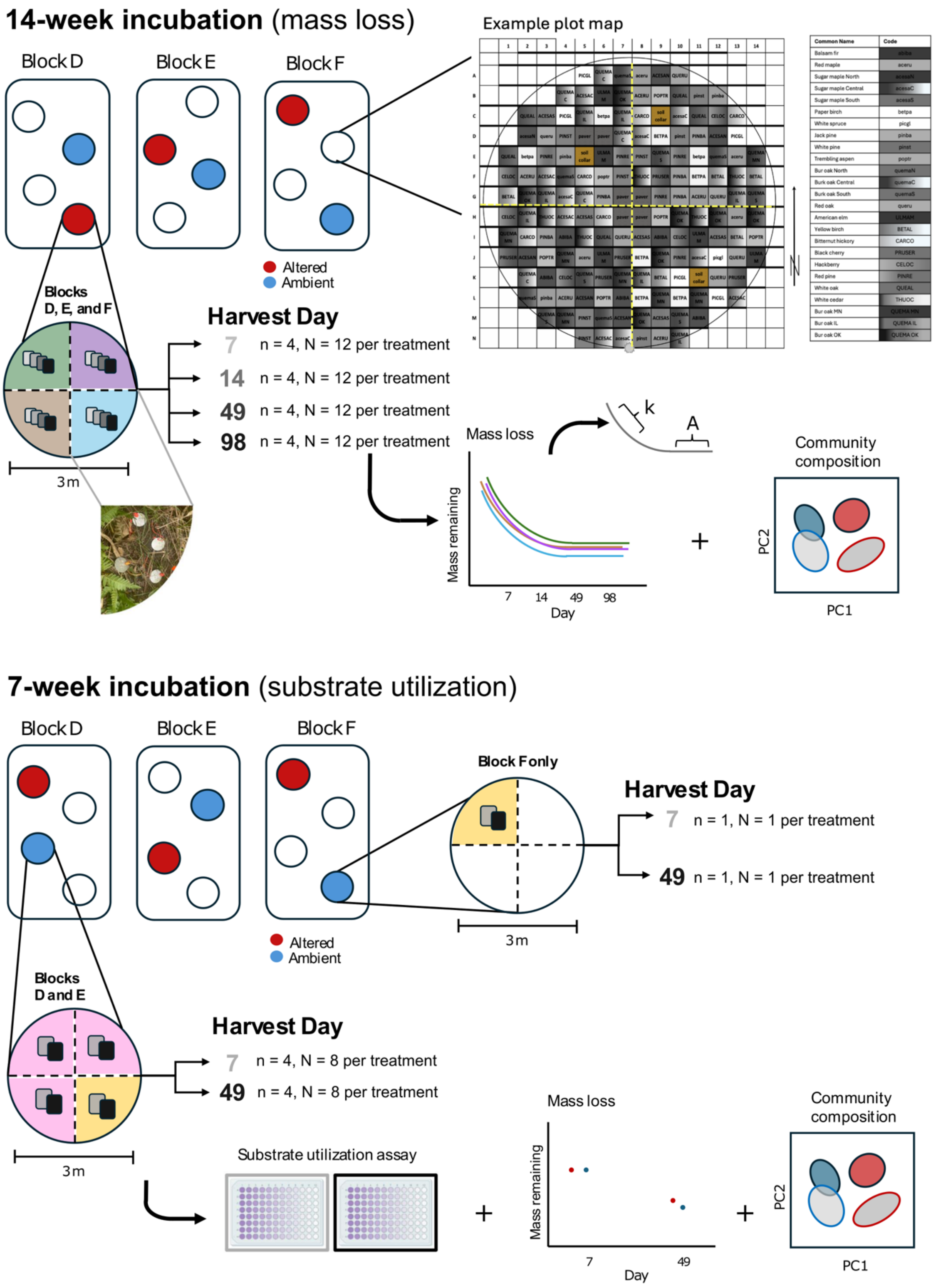

Supplemental Figure 1: Experimental design for the 14-week incubation and 7-week incubation. The 14-week incubation consisted of three separate blocks, each containing the two plot types (altered and ambient). White circles represent other plot types (not used in this study). A total of four harvest time points were conducted in the 14-week incubation, each represented with a different shade of gray (n = 4). Within the 3 m example plot, each color represents a quadrant, in which a unique model was run to obtain k and A values (N=12) for each treatment. An example plot map is represented in the center of the figure; each box represents a tree that has been planted in those coordinates. The legend of the trees planted is on the right of the map. The brown/orange boxes represent soil collars, which represent locations were soil respiration is taken (not presented in this study). The 7-week incubation, which was done to determine substrate utilization capacity, was conducted in two of the three blocks (blocks D and E). Bags were incubated in three out of four quadrants and were clustered in groups of two to account for two harvest points. An additional set of bags was deployed in the fourth quadrant of blocks D and E, and a quadrant of block F to assess measure microbial community composition and necromass mass loss.

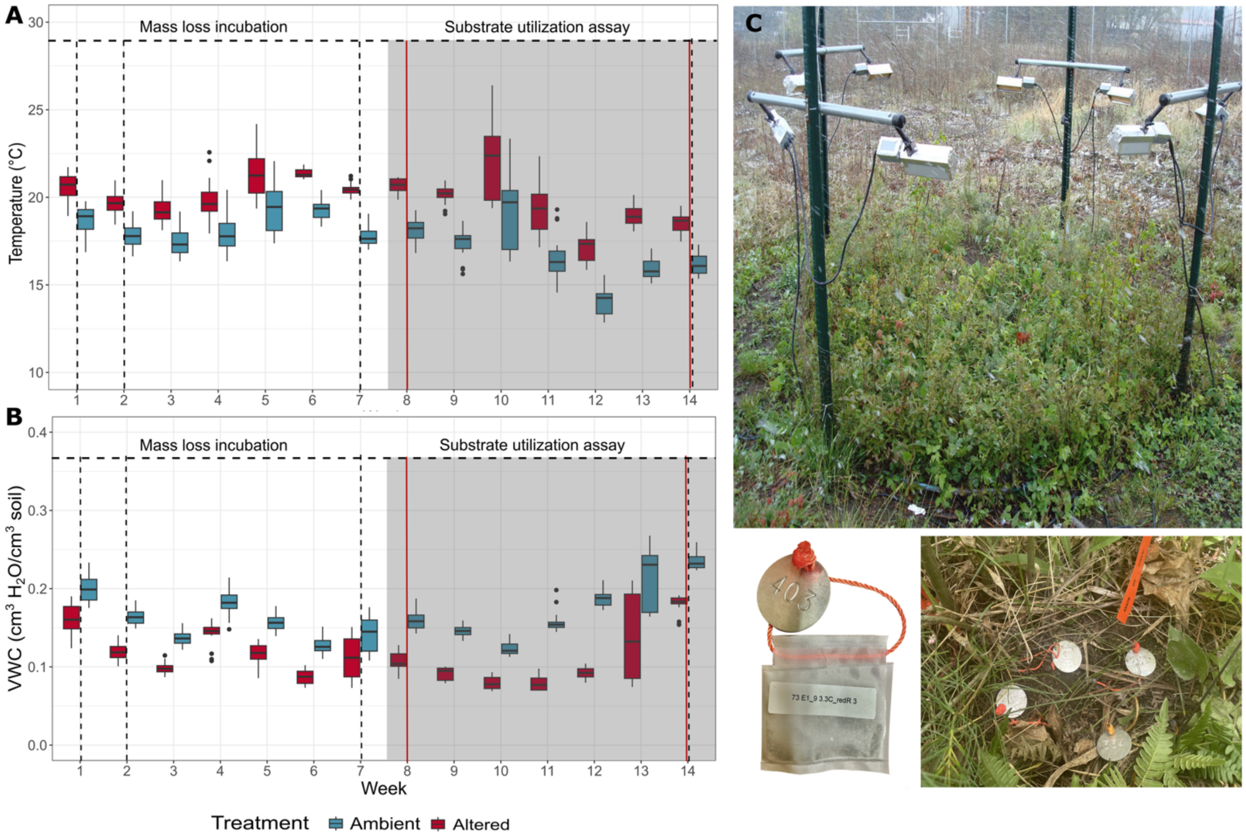

Supplemental Figure 2: Weekly temperature (A) and volumetric water content (B) trends over the period of the full experiment. Black dashed vertical lines represent harvest times of the necromass bags and red solid lines represents harvest times for the necromass bags used for the substrate utilization assays. Week 1 represents one week after initial deployment on June 28th, 2023, week 14 represents the last incubation harvest on October 4th, 2023, and week 7 (August 16th, 2023) was the deployment for necromass bags used for substrate utilization assays. Box plots contain median lines, with whiskers representing 10 and 90 quantiles. (C) Images of an altered experimental plot, with a cluster of buried necromass bags and an individual necromass bag pre-incubation.

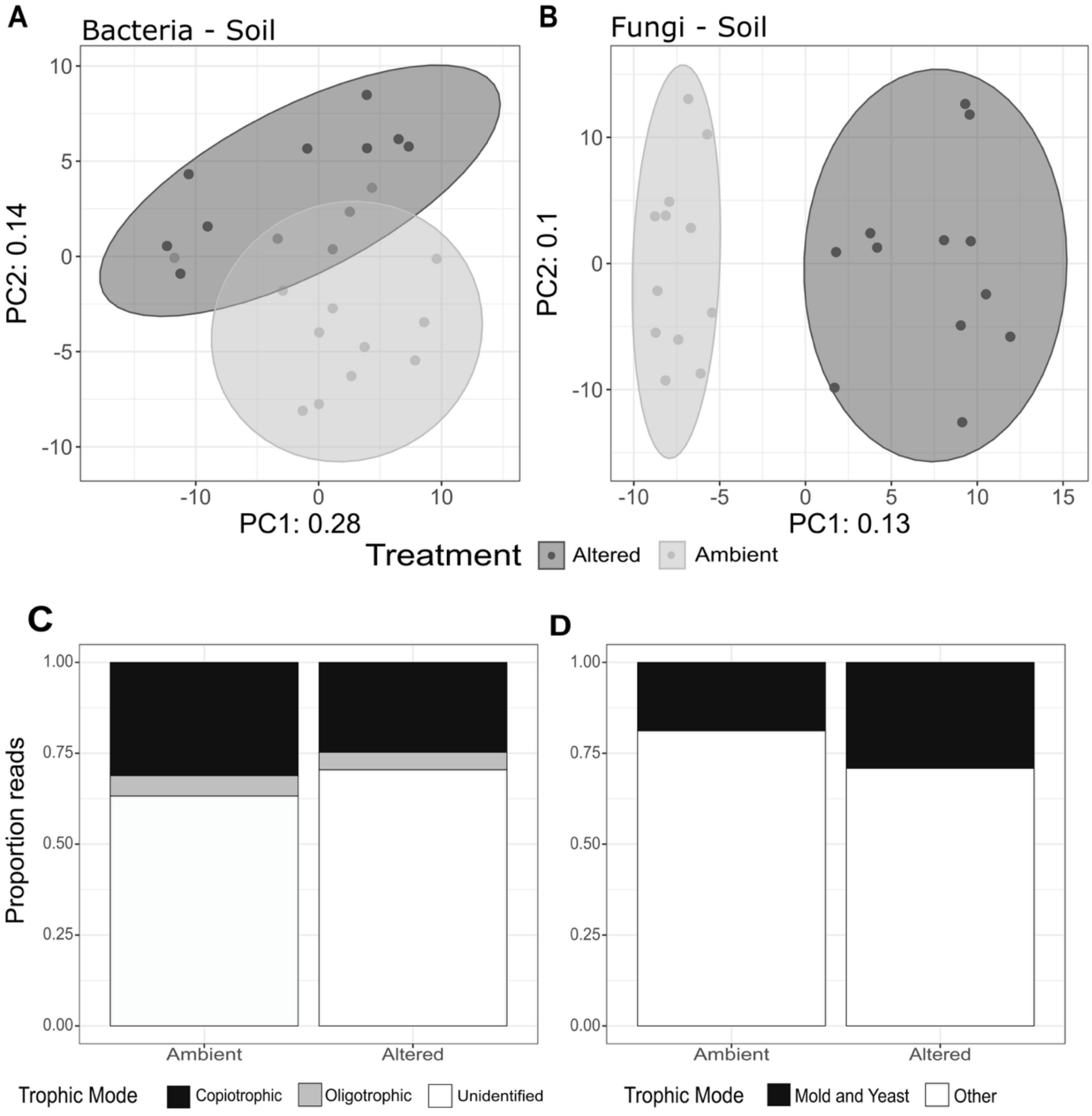

Supplemental Figure 3: Principal Components Analysis (PCA) for the bacterial (A) and fungal (B) communities in soil at the beginning of the incubation. Proportion of reads of bacteria (C) and fungi (D) found on soil classified by their trophic modes across treatments. Trophic modes for bacteria include copiotrophic (black), oligotrophic (gray), and unidentified (white), while trophic modes for fungi include mold and yeast (black) and other (white).

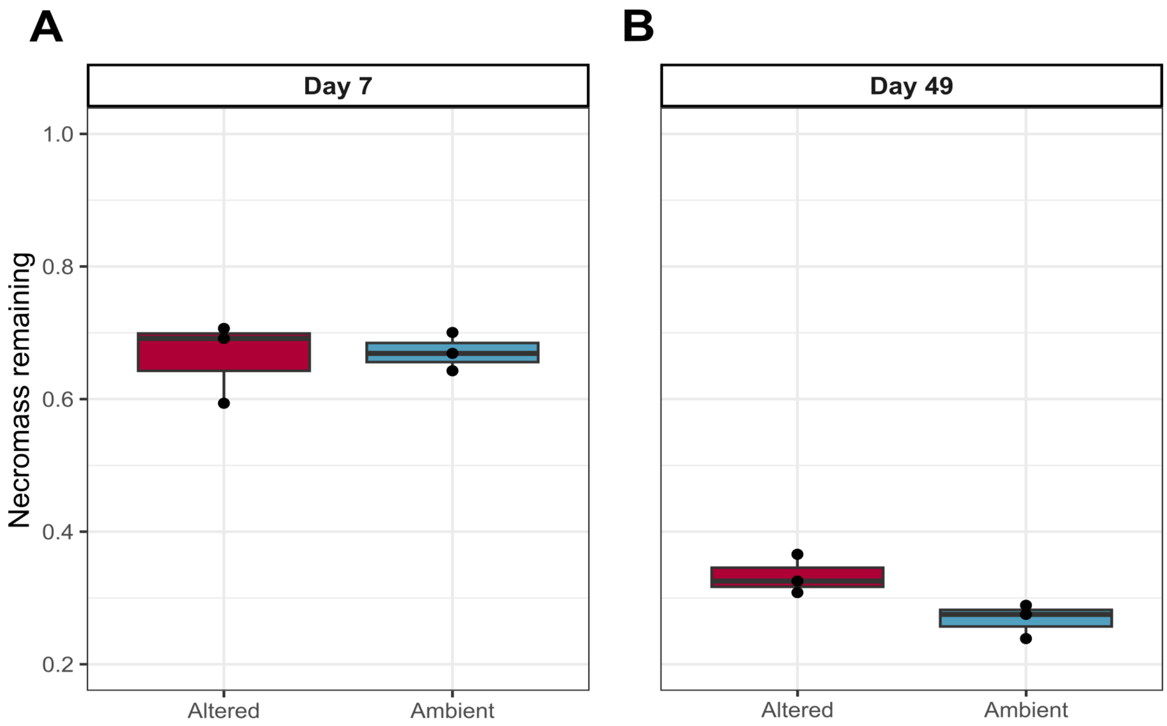

Supplemental Figure 4: Proportion of necromass mass remaining for the 7-week incubation after 7 days (A) and 49 days (B). Each point represents a single bag deployed in each block (n = 3, blocks D, E, and F) for each treatment condition.

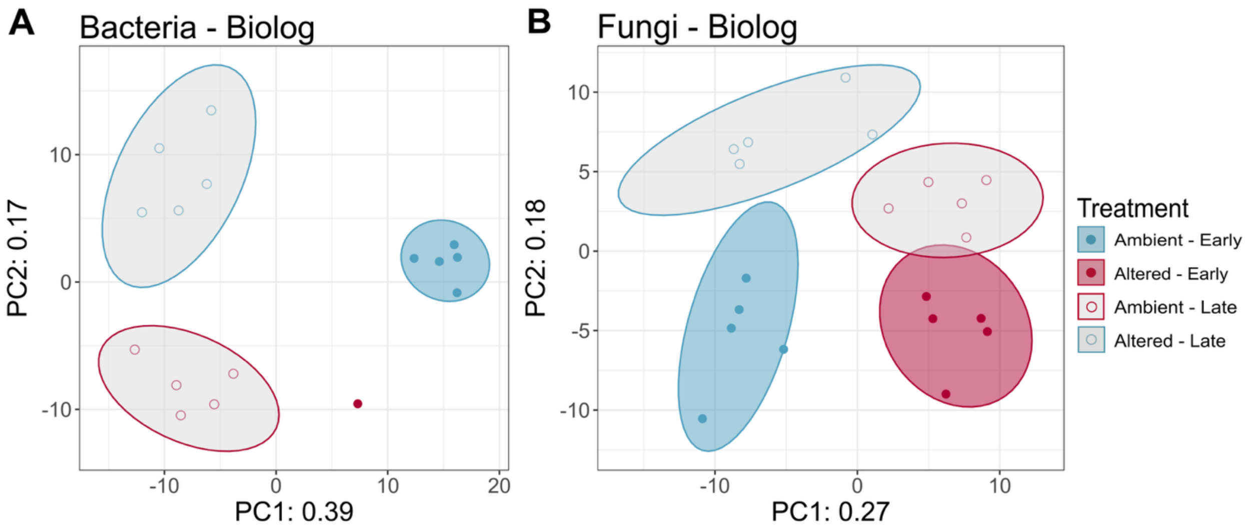

Supplemental Figure 5: Principial Components Analysis (PCA) for the bacterial (A) and fungal (D) communities present in the substrate utilization assay. Microbial communities for the ambient conditions are represented in blue and altered conditions in red. Different time periods are represented as "Early" for necromass incubation at day 7 and "Late" for necromass incubation at day 49. Only one point was captured during the early stage of decay under the altered conditions for bacteria because the center log transformation removed four samples which contained >80% zeros/unobserved in those samples. The five points under each condition here represent pooled samples used for substrate utilization (n=2, blocks D and E) and unpooled samples used for community composition and mass loss (n=3, blocks D, E, and F).

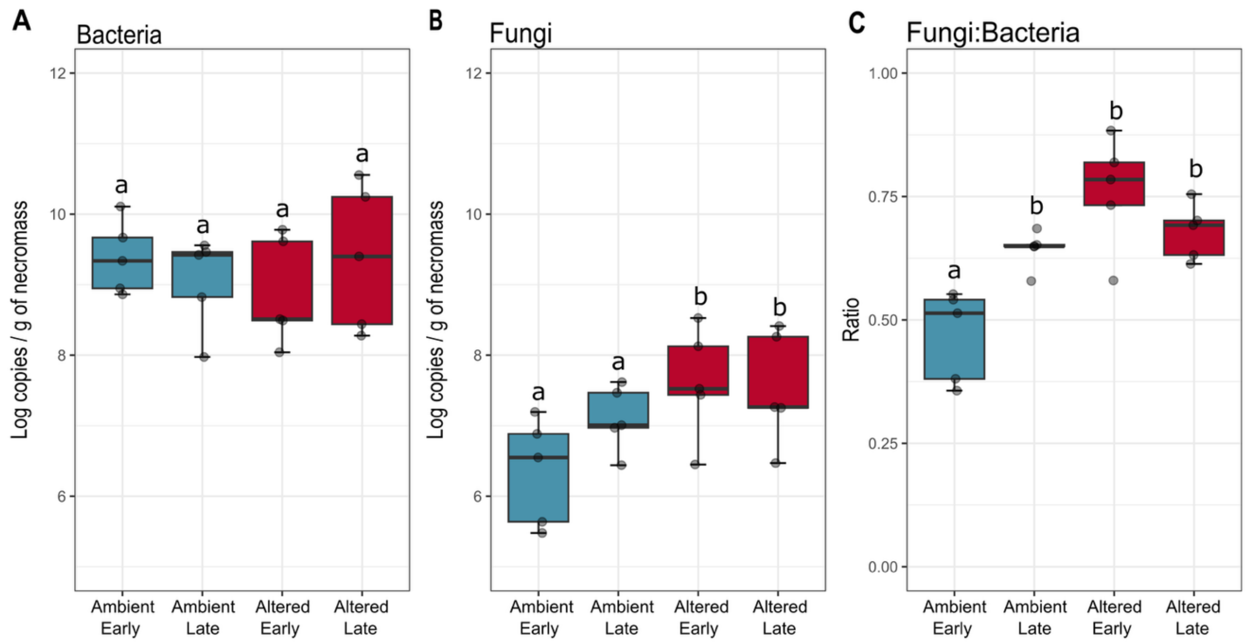

Supplemental Figure 6: Log number of 16S (bacteria) (A) and 18S (fungi) (B) gene copies obtained by qPCR on fungal necromass as well as fungal:bacterial gene copy ratios (C) across treatments for the 7-week incubation. Ambient plots are colored in blue and altered plots are colored in red. Significant differences between treatments across the two incubation times from Tukey's HSD test are indicated with different letters. Error bars represent ±1 standard deviation. The 5 points under each condition here represent pooled samples used for substrate utilization (n=2, blocks D and E) and unpooled samples used for community composition and mass loss (n=3, blocks D, E, and F).

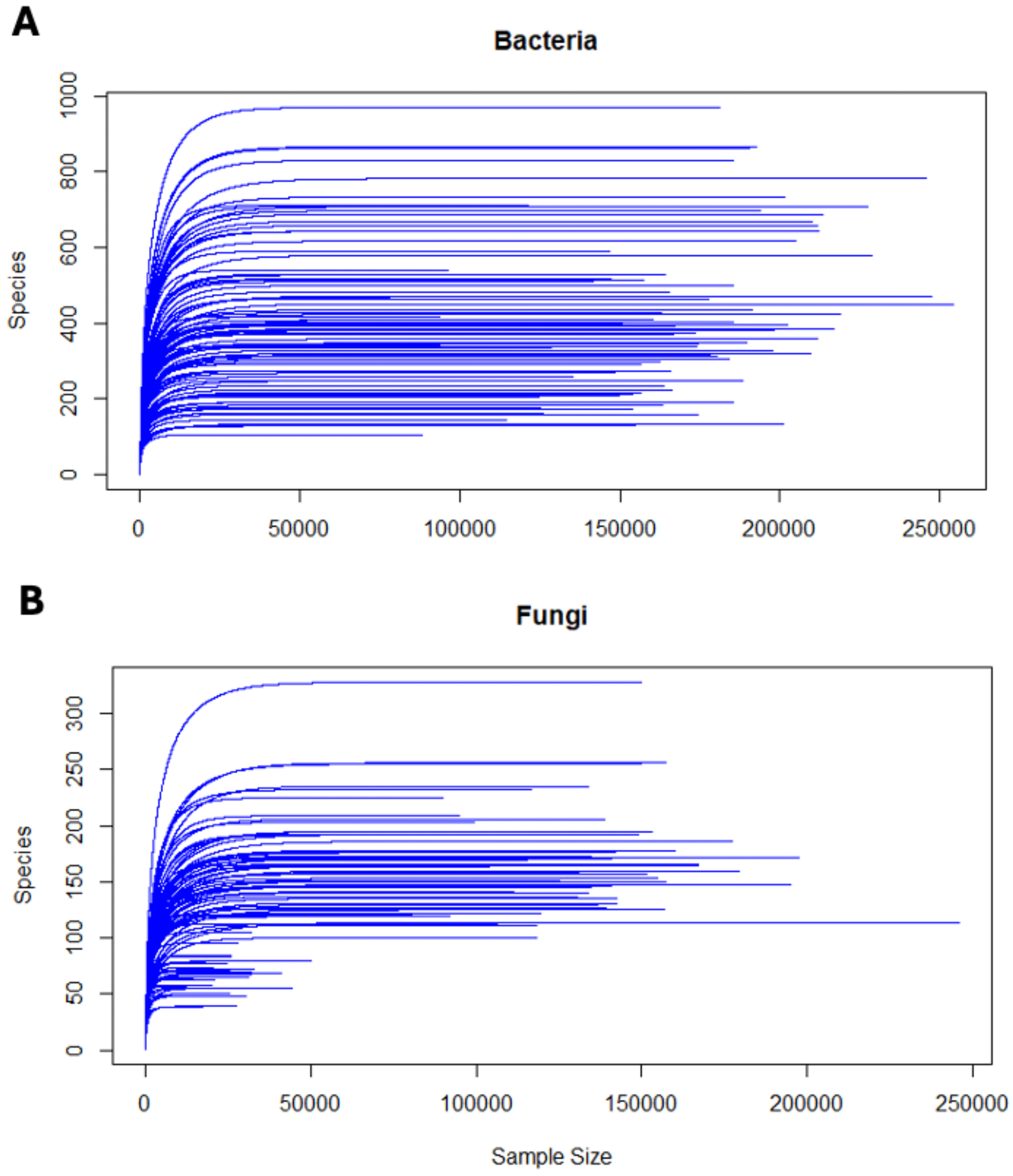

Supplementary Figure 7: Rarefaction curves of bacterial (A) and fungal (B) sequence reads for the samples of in the 14-week incubation.

Supplementary Tables

See Excel spreadsheet.
